# Physiologically based pharmacokinetic modeling for predicting irinotecan exposure in human body

**DOI:** 10.18632/oncotarget.18380

**Published:** 2017-06-06

**Authors:** Yingfang Fan, Najia Mansoor, Tasneem Ahmad, Rafeeq Alam Khan, Martin Czejka, Syed Sharib, Dong-Hua Yang, Mansoor Ahmed

**Affiliations:** ^1^ Department of Hepatobiliary Surgery, Zhujiang Hospital, Southern Medical University, Guangzhou 510280, China; ^2^ Department Pharmaceutical Sciences, College of Pharmacy and Health Sciences, St. John's University, Queens, NY 11439, USA; ^3^ Department of Pharmacology, Faculty of Pharmacy & Pharmaceutical Sciences, University of Karachi, Karachi 75270, Pakistan; ^4^ Pharma Professional Services, Karachi 75270, Pakistan; ^5^ Department of Clinical Pharmacy and Diagnostics, University of Vienna, A-1090 Vienna, Austria; ^6^ Department of Pharmaceutical Chemistry, Faculty of Pharmacy & Pharmaceutical Sciences, University of Karachi, Karachi 75270, Pakistan

**Keywords:** physiologically based pharmacokinetics (PBPK), irinotecan, colorectal cancer

## Abstract

Colorectal cancer is the third leading cause of cancer-related deaths in the United States. Treatment of colorectal cancer remains a challenge to clinicians as well as drug developers. Irinotecan, a Camptothecin derivative, is successfully used for the treatment of this rapidly progressing malignancy and finds its place in the first line of therapeutic agents. Irinotecan is also effective in treating SCLC, malignant glioma and pancreatic adenocarcinoma. However, its adverse effects limit its clinical application. Mainly metabolized by hepatic route, and excreted through biliary tract, this dug has been found to possess high variation in patients in its pharmacokinetic (PK) profile. Physiologically based pharmacokinetic (PBPK) models using compartmental approach have attained their position to foresee the possible PK behavior of different drugs before their administration to patients and such models have been proposed for several anticancer agents. In this work, we used WB-PBPK technology to develop a model in a population of tumor patients who used IV irinotecan therapy. This model depicted the concentration of drug and its pharmacologically active metabolite in human body over a specific period of time. Knowledge about pharmacokinetic parameters is extracted from this profile and the model is evaluated by the observed results of clinical study presented in literature. The predicted behavior of the drug by this approach is in good agreement with the observed results and can aid in further exploration of PK of irinotecan in cancer patients, especially in those concomitantly suffer from other morbidity.

## INTRODUCTION

Pharmacokinetic (PK) evaluation of anticancer agents is sparsely available in literature because of the highly inter individual variability exhibited by drugs. Anticancer drugs frequently show a narrow therapeutic index (TI), a fact that requires precise dosing in order to minimize toxicity while at the same time maintaining sufficient drug delivery to tumor cells needed for effective clinical activity. In recent years, physiologically based pharmacokinetic (PBPK) approaches have been successfully applied for the use of anticancer agents. PBPK modeling is based on an approach which starts with model building required both *in vitro* and *in vivo* data followed by model verification. Various PBPK models have been successfully used for some small molecular targeting drugs in predicting their exposure to human body and designing phase I clinical studies [1].

Early from the start of this century, the development of different models using PBPK modeling and simulation technique to describe detailed PK/PD data of multiple drugs is increasingly gaining attention from all aspects including academia, industry, and regulatory authorities and has now been established as an advanced approach for drug exposure-response analysis and clinical trial simulations [2, 3].

PBPK modeling and simulation technique possesses a huge potential and aids in efficient mechanistic understanding of pharmacokinetic as well as pharmacodynamic behavior of a drug and its metabolites. This understanding enables therapists to confidently make decisions regarding therapeutic strategies about clinical scenarios that have not yet been tested experimentally. Most importantly conducting population studies using PBPK approaches provides data that can be used to save time and resources otherwise required for experimental studies and to make experimental clinical trials confirmatory rather than exploratory.

In clinical practice, the required therapeutic doses of almost all anticancer agents are calculated on the basis of body surface area (BSA). However, it has been reported that the PK of many anticancer agents are not necessarily related to BSA [4]. Although the value of BSA-based dosing is frequently questioned [5], this approach is commonly practiced [6]. To identify the patient factors that involve in predicting drug exposure and its ultimate pharmacodynamic response, population-based evaluations via simulation approach are carried out [7], that are extremely useful in the development of appropriate dosing regimens. In 1998, PK and PK-PD model implementation had been suggested to revise the dosage of chemotherapeutic agents after measurement of drug concentration [8]. In the following decade, Evans et al. [9] reported that the 5-year survival in children with acute B-cell lymphoblastic leukemia improved from 66% to 76% by individualizing the dose of methotrexate based on individual patient's ability to clear the drug from plasma. A fully individualized methotrexate dosing in pediatric patients has also been suggested by other researchers further improving treatment outcomes [10]. Although a prospective clinical trial fully powered by modeling and simulation approach evaluating the outcomes predicted by this technique compared with standard care dosing system still awaits to be carried out, there are high expectations that this approach would be useful in near future to reduce toxicity while maintaining or even in some cases improving the treatment efficacy.

Usually, PK models are mainly based on mathematical mass balance equations that characterize drug absorption and disposition within the body. The PBPK models also consider anatomical and physiological realities, taking into account the various differences of distribution within and among organs in conjunction with their varying blood flows [11, 12]. A typical whole -body PBPK approach conceptualizes multiple compartments representing different organs of the body linked together by the arterial and venous routes of blood circulation.

Whole-body PBPK models have been reported in literature for several clinically used antineoplastic agents, such as methotrexate, 5-fluorouracil, adriamycin, doxorubicin, and for many newer compounds demonstrating antitumor activities such as everolimus and genistein [13, 14]. These models indicate how PBPK models can be useful in representing mechanistic drug-dependent features ultimately leading to more accurate predictions and in providing insights on physiological variables that can influence a drug disposition and whether concomitantly given drugs might interact with each other effecting the final outcome of the therapy.

Colorectal cancer is among one of the most common and deadly malignancies with an incidence of 1.2 million new cases per year globally and is estimated to be the third most common cause of cancer-related deaths in USA [15]. It has been considered as moderately resistant to chemotherapy. Though colonoscopic screening has considerably reduced the death rate of this disease in developed countries, patients in most under developed and developing countries are still diagnosed at advanced stages due to the expenses of colonoscopy added with poor access to health care.

**Figure 1:**
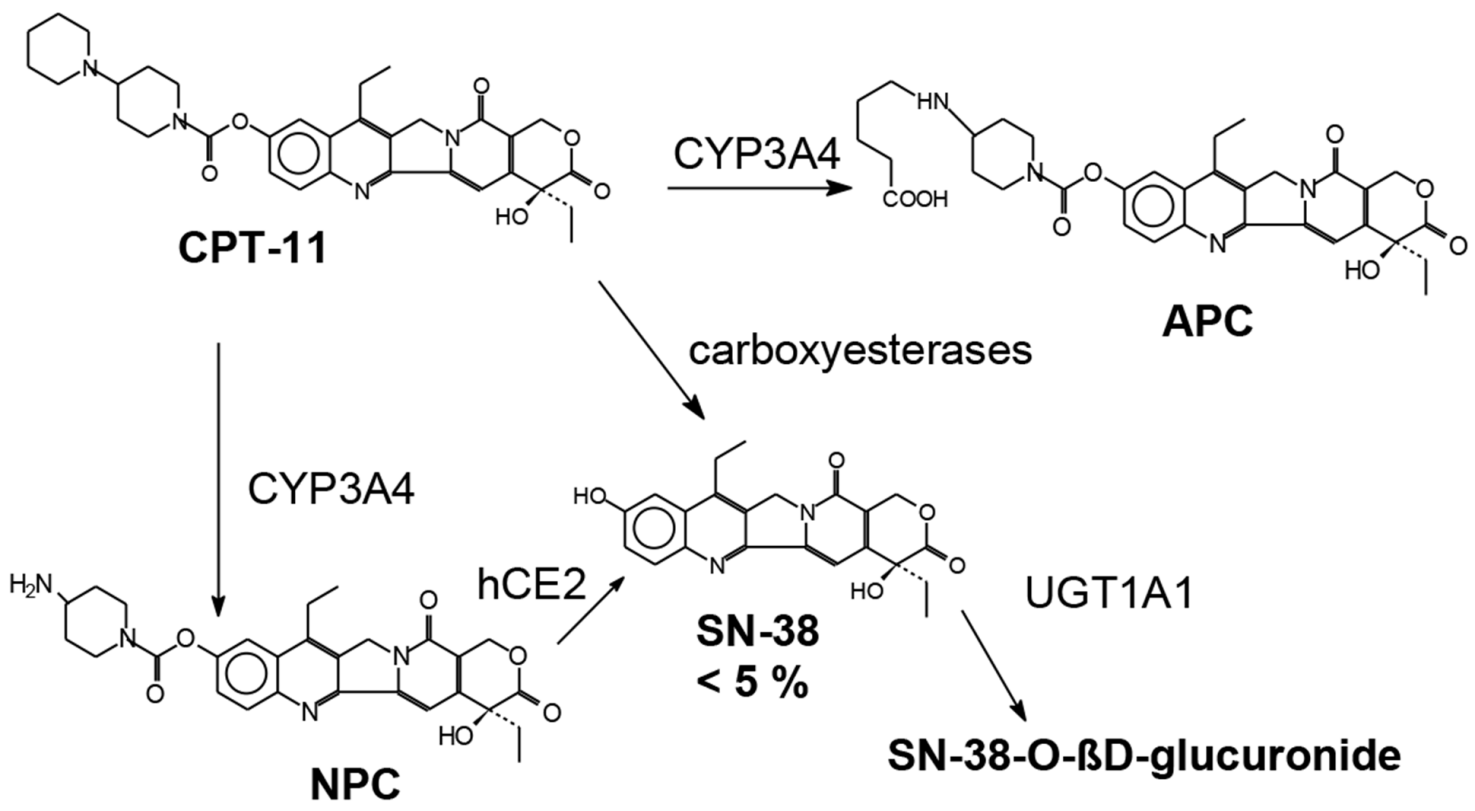
Chemical structure and metabolism of irinotecan

Irinotecan (CPT-11) is a camptothecin derivative which is metabolized to produce 7-ethyl-10-hydroxycamptothecin (SN-38) after carboxyl esterase-mediated breakdown [16] (Figure 1). SN-38 is the active metabolite of irinotecan [17] that inhibits topoisomerase I, a nuclear enzyme that is essentially required for relaxation of super coiled DNA [18] and is 100 -1000 times more potent than Itinotecan, inducing cytotoxic changes and finally causing apoptotic death of the malignant cells [19, 20]. Further inactivation and metabolism of SN-38 to SN-38G occurs through hepatic uridine diphosphate glucuronosyl transferase 1A1 (UGT1A1) [19].

CPT-11received FDA approval in 1998 for the treatment of metastatic colorectal carcinoma that has recurred or progressed following treatment with standard chemotherapy [21]. Avastin and CPT-11 combination therapy has shown rapid clinical as well as radiographic improvements in patients with relapsed malignant glioma. It has been reported that Topoisomerase I and II activities are significantly enhanced in malignant gliomas following DNA damage [22] by the single-agent irinotecan therapy. Irinotecan has also been shown to be effective in patients with extensive SCLC. New indication of Irinotecan has been reported in metastatic pancreatic ductal adenocarcinoma [23].

This paper presents the first detailed PBPK description of Irinotecan pharmacokinetics in human tumor patients by developing a PBPK model for irinotecan IV infusion of 350 mg for 30 minutes. The model describes the pharmacokinetic parameters considering realistic human parameters, such as blood flow indifferent organs and the tissue/blood partition.

## RESULTS

**Figure 2:**
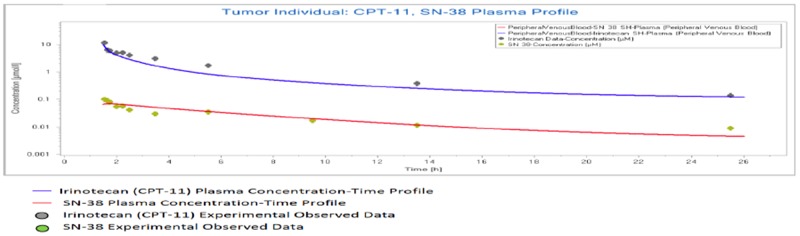
Population simulation result in PK-Sim Predicted plasma profile of CPT-11 and SN-38 in an individual with tumor.

**Figure 3:**
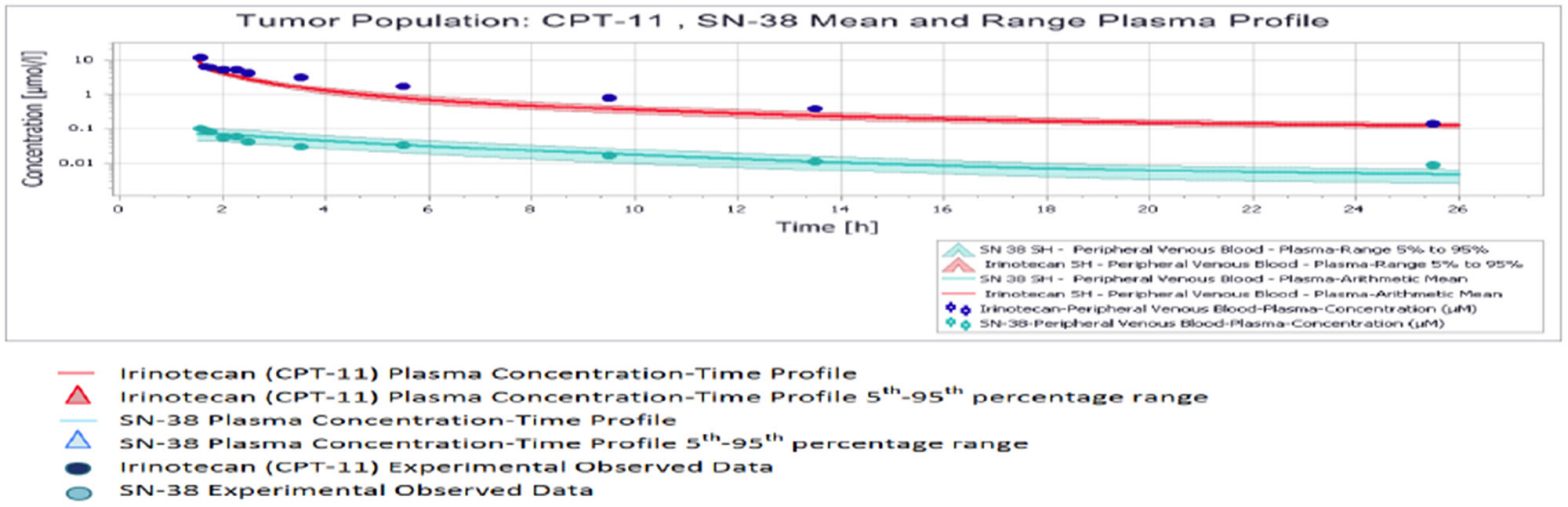
Mean and 5^th^-95^th^ percentile plasma profile of CPT-11 and SN-38 in the tumor population

**Figure 4:**
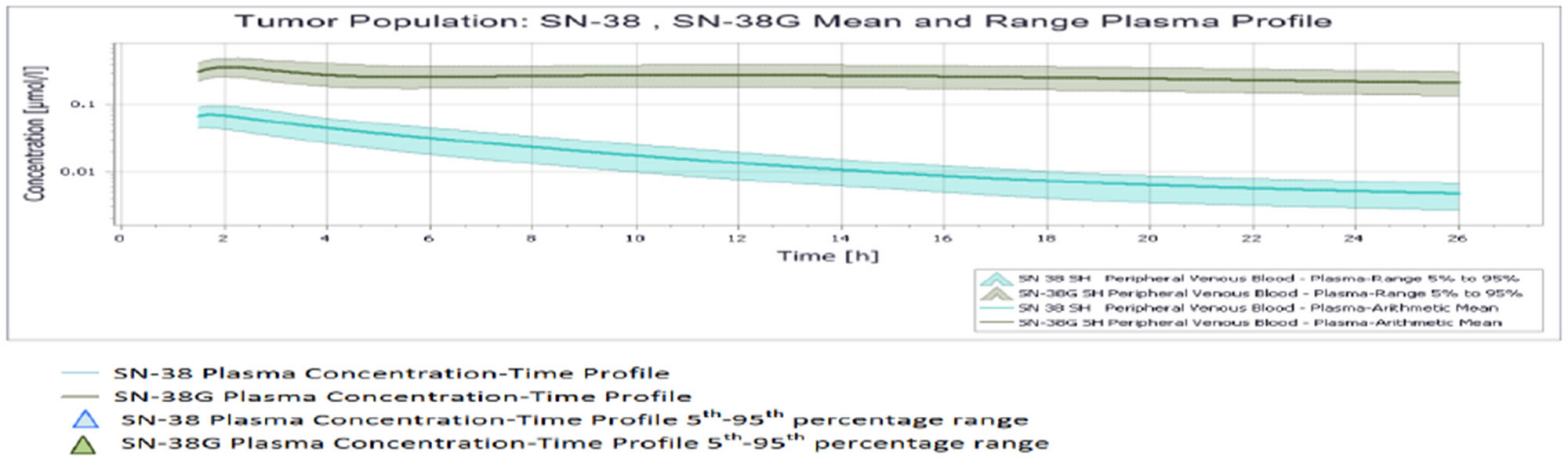
Mean and 5^th^-95^th^ Percentile Plasma Profile of SN-38 and SN-38 G in the tumor population

All results were depicted using standard Pk-Sim and MoBI graphical output. Numerical evaluation and visual predictive checks were adapted for comparison of predicted and clinically observed results. The graphical representation of plasma-concentration profile of CPT-11 and SN-38 in comparison to the experimentally observed data in our generated population of patients was shown in Figure 2, The PK analysis of these compounds in terms of the mean values of parameters are shown in Table 1, which are AUC 1296.06 μmol/ min/l, C_max_ 9.86 μmol/l. half life 29.21 h and the t_max_ 1.5 h. For irinotecan's active metabolite SN-38, the mean AUC, C_max_, t ½ and t_max_ values calculated for this population were 30.37 μmol/ min/l, 0.07 μmol/l., 19.47h and 1.7h respectively. The prediction of pharmacokinetics was enabled due to the incorporation of different physico-chemical properties. Lipophilicity (Log P), Fraction unbound of drug and PKa values have shown impact on the prediction of plasma concentration profile. Log P value determines the ability of drug to transport through membranes, Fraction unbound of drug lead to determination of volume of distribution of drug and PKa has a direct impact on the Lipophilicity and protein binding of the drug i.e. eventually related to drug pharmacokinetics [24]. The mean and 5^th^ to 95^th^ percentile profiles of CPT-11 and SN-38 are shown graphically in Figure 3. Figure 4 depicts the comparative plasma concentrations of SN-38 and water soluble SN-38G versus time. It is evident from Figures 3 and 4 that SN-38 is extensively metabolized by glucuronidated enzyme presenting greater concentration of SN-38G than SN-38 in plasma. Our physiologically based compartmental analysis method for predicting human PK yielded good predictive results and show that these pharmacokinetic profiles of irinotecan and SN-38 were in agreement with the observed data. These results indicated the ability of the model to describe irinotecan and its metabolite exposure in adults after IV application. Changing the pH environment of tumor tissue or blood plasma exhibited no detectable effects on pharmacokinetic parameters of parent drug or its metabolite as no difference of any degree was noted for AUC _tend_, C_max_, half life, plasma clearance, MRT, and volume of distribution of CPT-11 and SN-38 at different pH ranges of both blood plasma and tumor tissue.

**Table 1:** Tumor population pharmacokinetics (100 individuals): 50% female and 50% male

Parameters	Concentration mean irinotecan plasma levels	Concentration mean SN-38 plasma levels
AUC-tend (μmol* min/l)	1296.06	30.37
AUC-inf (μmol* min/l)	1612.57	38.36
AUC-inf-norm (μg* min/l)	NaN	NaN
AUC- tend -norm (μg* min/l)	NaN	NaN
Cmax(μmol/l)	9.86	0.07
Cmax norm (mg/l)	NaN	NaN
C-tend (μmol/l)	0.13	0.00474
Total body clearance (ml/min/kg)	NaN	NaN
% AUC (t last-∞)	0.2	0.21
MRT (h)	16.47	16.45
tmax (h)	1.5	1.7
Half life (h)	29.21	19.47
Vdplasma (ml/kg)	NaN	NaN
Vdssplasma (ml/kg)	NaN	NaN

## DISCUSSION

Malignant tumors often progress rapidly. It is important to make decisions for drug dose selection, adjustment and combination of different anticancer agents in a short period of time. Various anticancer agents are known to exhibit narrow therapeutic index and high subject variability. Experimental studies in cancer patient population to investigate the pharmacokinetics of drugs during drug development or after clinical application demand long periods of time, because of many hurdles such as less number of patient participation, patients’ frequent drop out from ongoing clinical research as a consequence of deteriorating general physical health owing to adverse effects of chemotherapeutic agents, and getting multiple blood samples in already debilitated patients with possible hematological abnormalities caused by chemotherapy, among others.

Modeling and simulation approaches make the envision of complex absorption, distribution, metabolism, and excretion outcomes of various drugs possible. These approaches can also predict profiles of parent drugs and their metabolite in various clinical scenarios. A growing interest in PBPK models is also the result of its utility in PK/PD-directed drug development [26, 27]. Simulations of virtual patient populations through these methods can save time and resources required by experimental studies and may play significant role in individualizing the dose of chemotherapeutic agents, increasing drug efficacy and desired consequences in every patient. These techniques also allow the simulation of virtual patient populations with altered physiological status such as obese/morbidly obese individuals and patients with renal impairment or liver cirrhosis.

Our present work is planned to provide a useful insight in the pharmacokinetic of IV infusion of Irinotecan in tumor patient population by the use of specialized physiologically based pharmacokinetic modeling technique. This work not only gives the plasma concentration profiles of parent drug irinotecan and its metabolite SN-38 but also provides their concentrations in tumor tissue which may provide opportunity for enhanced insight into pertinent PK thus aiding in design and adjustment of therapies.

CPT-11 and SN-38 has two isomeric forms: the open hydroxy-carboxylate form and the closed cyclic lactone form. Both isomeric forms are in an equilibrium state that depends on the pH value of the medium (blood, plasma, tissue, etc). At pH values above 7.0, the equilibrium shifts towards the side of the lactone ring, whereas at low pH values (about 4.0 ˆ 6.0), the ring opened hydroxy carboxylate dominates with only small amount of the lactone form. The cyclic lactone form of SN-38 represents the pharmacologic active molecule that is capable to inhibit topoisomerase while the carboxylate form is responsible for the toxic side effects of SN-38, especially diarrhea. Tumor tissue has a lower pH than healthy tissue or plasma hence the equilibrium shifts to the side of the hydroxy carboxylate form in tumor tissue.

However, our simulated pharmacokinetic profiles gained at different pH values of blood plasma and tumor tissues interprets that pH change in tissues or plasma does not seem to have any effect on the pharmacokinetic behavior of the CPT-11 and SN-38 though it is of significant pharmacodynamic importance.

Using this model for different races of humans can explore differences that may be present among them because of possible differences in their genetic makeup. It has been shown that CPT-11 administration is associated with hepatotoxicity, an effect that is compounded by baseline obesity. Patients with a BMI index of >25 were found twice as much susceptible to developing liver toxicity than patients with BMI index of 25 or lower. A growing interest in PBPK modeling techniques is their potentials of confirming these types of findings or to make predictions on drug behavior in different morbidities compounded together, conditions that are frequently encountered during clinical practice, in a relatively shorter period of time.

## CONCLUSIONS

Irinotecan is a potent anticancer agent being the first line agent in the treatment of multiple oncologic conditions. As with other chemotherapeutic agents, the severity and incidence of adverse effects limit its clinical application. As PBPK modeling has successfully been applied to assess various untested clinical situations, the model we developed to understand the behavior of irinotecan may be used to gain knowledge about potential exposure changes of this drug in cancer patients with various co-morbid conditions such as organ failure or metabolic disorders. In the future, with the availability of more and more innovative techniques for measuring tissue drug concentrations, this type of predictive tissue-based PK modeling may supervene and integrate to medical fields for delivering better treatment to patients with dose individualization according to their health status and increasing the success of chemotherapy.

## MATERIALS AND METHODS

The PBPK modeling was established in computer using the software Pk-Sim 6.0 and MoBi 6.0. Initially, a virtual whole body PBPK model was developed for an adult of European race having biometrical values set according to the ones used in experimental study by Rivoryet al. [25]. A literature survey was carried out to collect the physicochemical attributes of irinotecan for molecular modeling that are shown in Table 2. Irinotecan (CPT-11) shows complex metabolism, and efforts have been put to incorporate complete metabolic and transport processes for CPT-11 and SN-38. The metabolic conversion of CPT-11 to SN-38 is mainly through carboxyl esterase. However, CYP3A4 is also involved indirectly in this process. UGT enzyme family specifically UGT1A1, UGT1A6, and UGT1A9 further metabolize SN-38 to a more water soluble product SN-38 G. CPT-11 and SN-38 movement from cell to interstitial space and through bile to intestine is carried out by various transport proteins which are incorporated in this model development.

**Table 2:** Physicochemical properties of CPT-11 and SN-38

Parameter		Value		Reference	
Compound		Irinotecan	SN-38	Irinotecan	SN-38
pKa	Acid	11.71	9.68	Drug bank	
	Base	9.47	3.91		
Lipophilicity log P		2.78 log units	1.87 log units	ChemAxon	ALOGPS
Solubility/pH		0.11mg/ml at 7 pH	0.29 mg/ml at 7pH	Drug Bank	
Fraction unbound (*Fup*)		0.32	0.05	Drug Bank	
Dose		350 mg/m2 or 750 mg	Nil	Rivory, Laurent P., et al	
Molecular weight		677.10	392.40	Drug Bank	
Effective molecular weight		655.10	392.40	Drug Bank	
Renal clearance		GFR fraction =1	GFR fraction =1		
Biliary clearance (specific clearance)		2.70 ml/min/kg	2.70 ml/min/kg	Optimization for experimental plasma profile	

Our Irinotecan PBPK modeling uses previously determined standard human physiological and anatomical data set for the organ volumes and blood flows. As irinotecan is eliminated through biliary route, specific biliary clearance is mentioned in the model development and kinetics of specific enzymes were incorporated in the building blocks and the simulation was created by linking together the individual's data and molecular data. The administration dose was set as 350 mg of Irinotecan intravenous infusion run for 30 minutes according to the study reported in literature [25]. Plasma concentration-time profile of irinotecan in this virtual individual was attained by running the simulation. This predicted profile was evaluated by applying it to the experimental data of Rivoryet al [25]. PK parameters of experimental study are shown in Table 3. Software MoBi works with PkSim in synergy and was used for tumor incorporation in this individual.

**Table 3:** Observed experimental data on the time (h) vs. plasma concentration (μmol. L) of irinotecan (CPT-11) and SN-38 after 350mg/m^2^ irinotecan infusion

Time (hours)	Irinotecan conc. umol/l	SN-38 conc. umol/l
1.56	11.6	0.1
1.66	6.25	0.09
1.75	5.95	0.08
2	4.98	0.058
2.25	5.05	0.055
2.5	4.12	0.042
3.5	3.05	0.03
5.5	1.72	0.035
9.5	0.8	0.017
13.5	0.38	0.0115
25.5	0.14	0.009

A population of 100 individuals comprising of both male and female patients was created based on the individuals’ Irinotecan and SN-38 median plasma concentration-time profile. The 5th and 95^th^ percentile range was obtained through population simulation using Pk-Sim. Finally, Pharmacokinetic parameters were obtained for the resultant profiles and are demonstrated in Tables 4 and 5. Furthermore, as tumor tissue has been reported to have a lower pH in comparison with the blood plasma, pharmacokinetic profiles of CPT-11 and SN-38 were also predicted for varying pH ranges of tumor tissue and blood plasma in order to evaluate the effect of these changes on pharmacokinetics of CPT-11 and SN-38. Different pH values between the ranges of 6.0 and 7.5 were selected for tumor tissue and blood plasma alternatively while keeping one of these at a constant value. Certain PK parameters such as AUC _t-end_, C_max_, half life, plasma clearance, MRT, and volume of distribution were predicted at these pH environments.

**Table 4:** Comparison of population median plasma profile and experimental plasma profile pharmacokinetics

Parameters	Population median CPT-11 plasma conc- profile	CPT-11 experimental data	Predictive error (%)
AUC_tEnd [μmol*min/l]	1296.06	1504.96	13.88
Cmax μmol/l	9.86	11.6	15
Tmax [h]	1.5	1.56	3.846
Half life [h]	29.21	Infinity	__

**Table 5:** Comparison of population median plasma profile and experimental plasma profile pharmacokinetics

Parameters	Population Median SN 38 Plasma Conc- Profile	SN-38 experimental data	Prediction error (%)
AUC_tEnd [μmol*min/l]	30.37	26.74	13.57
Cmax μmol/l	0.10	0.10	0.00
Tmax [h]	1.7	1.56	8.97
Half life [h]	19.47	Infinity	__
